# HPV-mediated regulation of SMAD4 modulates the DNA damage response in head and neck cancer

**DOI:** 10.1186/s13046-022-02258-9

**Published:** 2022-02-10

**Authors:** Simona Citro, Claudia Miccolo, Alessandro Medda, Lavinia Ghiani, Marta Tagliabue, Mohssen Ansarin, Susanna Chiocca

**Affiliations:** 1 Department of Experimental Oncology, IEO, European Institute of Oncology IRCCS, IEO Campus, Via Adamello 16, 20139 Milan, Italy; 2 Division of Otolaryngology Head & Neck Surgery, IEO, European Institute of Oncology IRCCS, 20141 Milan, Italy; 3 Department of Biomedical Sciences, University of Sassari, Sassari, Italy, University of Sassari, Sassari, Italy

**Keywords:** Head and neck cancer, Squamous cell carcinoma, SMAD4, HPV, Ubiquitin, βTRCP1, DNA damage response, p63, p53, Virus

## Abstract

**Background:**

Head and Neck cancer (HNC) is a fatal malignancy with poor prognosis. Human Papillomavirus (HPV) infection is becoming the prominent cause of HNC in the western world, and studying the molecular mechanisms underlying its action in cancers is key towards targeted therapy. To replicate, HPV regulates the host DNA damage repair (DDR) pathway. SMAD4 is also involved in the regulation of the DDR machinery and likely plays important role in maintaining cell viability upon genotoxic stress. In this study, we investigated the role of HPV in the upregulation of SMAD4 to control the DDR response and facilitate its lifecycle.

**Methods:**

SMAD4, Rad51 and CHK1 expression was assessed in HPV-positive and HPV-negative HNC using TCGA data, a panel of 14 HNC cell lines and 8 fresh tumour tissue samples from HNC patients. HPV16 expression was modulated by E6/E7 siRNA knock-down or transduction in HPV-positive HNC cell lines and Human Primary keratinocytes respectively. SMAD4 half-life was assessed by cycloheximide treatment in HNC cell lines, together with βTRCP1-dependent SMAD4 ubiquitination. SMAD4 siRNA knock-down was used to determine its role in HPV-mediated regulation of DDR machinery and to assess cisplatin sensitivity in HPV-positive HNC cell lines.

**Results:**

We found that HPV increases SMAD4 expression is both HPV-positive HNC tumours and cell lines, impairing its degradation which is mediated by the E3 ubiquitin ligase βTRCP1. SMAD4 expression highly correlates with the expression of two main players of the DDR pathway, CHK1 and Rad51, which expression is also upregulated by the presence of HPV. In particular, we demonstrate that HPV stabilizes SMAD4 to increase CHK1 and Rad51 expression. In addition, SMAD4-deficient HPV-positive cells have increased sensitivity to cisplatin treatment.

**Conclusions:**

Our results give a clear molecular mechanism at the basis of HPV regulation of the DDR pathway. In particular, we show how HPV stabilizes SMAD4 to promote DDR protein expression, which may be used to facilitate viral replication and HNC onset. Moreover, we found that SMAD4 silencing in HPV-positive HNC cell lines increases sensitivity to cisplatin treatment, suggesting that HPV-positive HNC with low SMAD4 expression may be preferentially susceptible to similar treatments.

**Supplementary Information:**

The online version contains supplementary material available at 10.1186/s13046-022-02258-9.

## Background

SMAD4, also called SMAD family member 4, Mothers against decapentaplegic homolog 4, or DPC4 (Deleted in Pancreatic Cancer-4) is a central mediator of TGF-β signalling by forming complexes with receptor-activated SMADs (SMAD-2 and -3). The SMAD complexes then translocate to the nucleus and regulate the expression of genes involved in many cancer-related processes such as proliferation, apoptosis and inflammation [1].

Reduced SMAD4 expression is common in tumours and occurs through a combination of mutations, copy loss and transcriptional downregulation [2, 3]. Reduced SMAD4 immunostaining has been associated with reduced survival in lung and pancreatic cancer [4, 5]. SMAD4 loss in animal models initiates tumour formation [3, 6], promotes the progression of oncogene-initiated lesions [7, 8], and stimulates the development of metastases [4, 9].

The loss of SMAD4 plays a crucial role in the response to DNA damage leading to increased genomic instability. This is very prominent in squamous cell carcinoma of the skin and of the Head and Neck cancer (HNSCC or HNC) and suggests a distinct role of SMAD4 in the progression of various types of tumours [6, 10].

HNC is the sixth most common cancer worldwide [11] with a 5-year survival of 50–60%. The use of tobacco and the excessive alcohol consumption are the most important risk factors so far identified and they also seem to have a synergistic effect. A subgroup of HNC, particularly those of the oropharynx, is caused by infection with high-risk types of human papillomavirus (HPV). In the western world, despite the incidence of HNC has been slowly declining in the past decade, probably due to a decrease in the prevalence of smoking, oropharyngeal cancers are becoming more prevalent. This may be related to an increase in oropharyngeal HPV infections and recent studies revealed that HPV-16 is mostly involved in these tumours. HPV-positive tumours form a distinct group within HNC with different aetiological factors.

The tumours are different at the molecular level impacting on the clinical outcome: in general, HPV-related HNC has a more favourable prognosis [12]. Loss of heterozygosity (LOH) at the SMAD4 locus has been reported in approximately 50% of human HNC [13]. The higher frequency of SMAD4 loss at the mRNA and protein levels compared to the percentage of LOH suggests that other epigenetic, posttranscriptional or posttranslational modifications contribute to reduced SMAD4 expression in HNC. In particular, published TCGA data analysis [14] showed decreased expression of SMAD4 mRNA expression in HPV-negative compared to HPV-positive HNC tumours.

The productive life cycle of high-risk HPVs is dependent on epithelial differentiation and on activation of host DNA repair pathways that also play critical roles in the development of many cancers. Many oncogenic viruses express proteins that affect the cell cycle and DNA damage repair regulatory pathways. Several studies have shown that during genome amplification, HPV proteins interact with different components of the cellular DNA repair machinery to activate or downregulate the expression or activity of factors of the ATM (ataxia-telangiectasia mutated) and ATR (ATM- and Rad3-Related) pathways. Importantly, many proteins from ATM and ATR pathways colocalize with HPV replication sites, further supporting the role of these factors in the HPV life cycle [15, 16].

Here, we report that HPV-positive HNC cell lines and tumours show increased expression of SMAD4 compared to the HPV-negative ones. This increased expression seems to be due to deficiency in SMAD4 degradation in HPV-positive cell lines, due to impaired ubiquitination by the βTRCP1 E3 ligase. Moreover, we demonstrate how HPV-mediated activation of the DNA damage regulatory pathway is dependent on SMAD4 expression and affects DNA-damaging drug sensitivity.

## Materials and methods

### Cell culture

HNC cell lines, acquired from different sources [17], were accurately described and cited in our previous studies [18, 19]. Cells were grown in Dulbecco’s modified Eagle’s medium (Euroclone) supplemented with antibiotics, 2 mM L-glutamine, 10% FBS (Fisher Scientific) and non-essential amino-acids (NEAA). Every 6 months all cell lines were authenticated by short tandem repeat profiling and tested for mycoplasma contamination. Skin biopsies were collected via standardized operative procedures approved by European Institute of Oncology Ethical Board. Informed consent was obtained from all patients. Adult human epidermal keratinocytes (HKs) were prepared and maintained as previously described [20, 21]. Primary cultures of the isolated cells were then maintained in Keratinocyte Serum-Free Medium (KSFM; Gibco) containing bovine pituitary extract (BPE, 30 μg/mL; Gibco) and epidermal growth factor (EGF, 0.2 ng/mL; Gibco). Cells from passages 2–5 were used for the experiments. All cells were cultured at 37 °C in a 5% CO2 buffered incubator. MG132 and cycloheximide were purchased from Sigma.

### Tissue samples

Fresh tumours and adjacent normal tissue samples from HNC patients were separately collected upon surgery under IEO Biobank approval and preserved at -80C degrees. Samples were then snap-frozen in liquid nitrogen and processed for RNA and protein extraction using AllPrep DNA/RNA/protein kit (Quiagen) following manufacturers’ instruction. Samples were collected from 5 HPV-negative and 3-HPV-positive HNC patients, were all derived from primary tumours except for one relapse, and from male patients except for one female patient. Samples were collected mainly from the oral cavity and the tongue, except for one that was collected from the pharynx.

### PCR amplification and PCR product directly sequencing

All encoding exons and flanking intronic sequences of SMAD4 were analysed by Sanger sequencing. Genomic DNAs (40 ng per reaction) from each cell line (UMSCC10A, UMSCC23 and UMSCC28) were amplified with primers designed for amplification of all encoding exons and around 50 base pairs of flanking intronic sequences of SMAD4. Amplified genomic DNA fragments were directly sequenced utilizing the same forward or reverse primers used in the original PCR amplification. All sequencing was performed on the Applied Biosystems TM 3500 Dx Genetic Analyzer capillary automated sequencers at the Cogenthech facility (Milan).

### Transduction, transfection and plasmids

pLXSN HPV16 E6/E7, pLXSN HPV11 E6/E7 and empty vector were previously described [21], pSuper Retro shp53 was a gift from M. Tommasino (International Agency for Research on Cancer). For retroviral transduction, plasmids were transfected into Phoenix Ampho cells by calcium-phosphate method. The following day, HKs or HNC cell lines were transduced with retroviral supernatants for 6 h at 37 °C for 2 days and selected with G-418 Sulfate (Gibco) or Puromycin for respectively 1 week or 3 days. HNC cell lines were transfected with Lipofectamine 2000 (Thermo Fisher Scientific), following manufacturers’ instruction for both siRNA oligos: siLuciferase (siLUC), E6/E7 siRNAs [21], SMAD4 (5′-GGUGAUGUUUGGGUCAGGUGCCUUATT-3′), ΔNp63α (5′-ACAAUGCCCAGACUCAAUUdTT-3′) and βTRCP1 (5′-GAUAAUACCAGAGAAGAAUdTT-3′), and plasmids: pcDNA3-βTRCP1-Flag [22] and pCMV p53 [23]. SMAD4 or Luciferase siRNA were transfected into HKs using RNAiMAX reagent (Thermo Fisher Scientific), following manufacturers’ instruction.

### Cell lysis and Western blotting

Cells were lysed in either a sodium dodecyl sulfate (SDS) lysis buffer: a 1:3 mixture of buffer I (5% SDS, 0.15 M Tris-HCl [pH 6.8], 30% glycerol) and buffer II (25 mM Tris-HCl [pH 8.3], 50 mM NaCl, 0.5% NP-40, 0.1% SDS, 1 mM EDTA) or Urea 8 M, containing 0.5 mM N-ethylmaleimide (NEM), 0.5 mM NaF and 2 mM sodium Na_3_VO_4_ and protease inhibitors (Sigma). After lysis, an equal amount of protein for each sample was resuspended in denaturing sample loading buffer, separated on SDS-PAGE gel and immunoblotted with the indicated antibodies. The following antibodies were used: SMAD4, CHK1, p-CHK1 and βTRCP1 (Cell Signaling Technology), p63, pH2Ax, Flag and GAPDH (Abcam), HPV16 E7, p53, H3 and Rad51 (Santa Cruz Biotechnology), and Vinculin (Merck Millipore). Membranes were then incubated with the appropriate horseradish peroxidase (HRP) secondary antibodies and the signal was acquired with Chemidoc (Bio-Rad). Densitometric analysis of the intensity of the protein bands were preformed using ImageJ software (Rasband W.S., ImageJ, U. S. National Institutes of Health), and standardized to the housekeeper levels.

### Statistical analysis of TCGA data

RSEM normalized expression data was extracted from the TCGA PanCancer Atlas on the cBioPortal [24, 25]. Patient samples with known HPV status were grouped as HPV+ and HPV-. This resulted in 72 HPV+ and 415 HPV-samples with data available for the HNC gene expression analysis. Boxplot comparisons of gene expression and statistical analysis was performed using GraphPad Prism v9.0 (Graphpad Software, Inc., San Diego, California, USA).

### RT-qPCR

RNA was extracted from cells with the Quick-RNA MiniPrep kit (Zymo research). cDNA was generated by reverse transcription-PCR with Reverse Transcriptase (Promega). Relative levels of specific mRNAs were determined with the Fast SYBR Green detection chemistry system (Applied Biosystem). All PCR reactions were performed with a QuantStudio 6 Fast Real-Time PCR system (Thermo Fisher Scientific). Ribosomal Phosphoprotein (RpP0) was used as a house-keeper gene for normalization.

### Immunoprecipitation

HNC cell lines were lysed in E1A lysis buffer (50 mM HEPES pH 7, 250 mM NaCl, 0.1% NP-40, proteases inhibitors, 0.5 mM NEM, 0.5 mM NaF and 2 mM Na_3_VO_4_) for co-immunoprecipitation and in SDS buffer to detect SMAD4 ubiquitination. Immunoprecipitation was performed in E1A buffer (50 mM HEPES pH 7, 250 mM NaCl, 0.1% NP-40, proteases inhibitors, 0.5 mM NEM, 0.5 mM NaF and 2 mM Na_3_VO_4_) using protein extracts incubated with the indicated antibodies o/n at 4 °C. SDS Samples were loaded on SDS-PAGE gel and immunoblotted with the indicated antibodies. Total extracts (input) were loaded as control.

### Confocal microscopy

Cells were seeded on coverslips and fixed with 4% PFA. Anti-Rad51 Rabbit and anti-pH2Ax mouse were incubated o/n at 4 °C. Goat anti-rabbit Alexa 488 was used as secondary antibody and nuclei were stained with DAPI. Coverslips were then mounted and images were taken using a Leica SP8 AOBS confocal microscopy.

### Cell viability assay

UDSCC2 cell line was transfected with specific siRNA against SMAD4 or Luciferase as control. 24 h after transfection, cells were seeded into 96well plates (7000 cells/well) and 24 h later were treated with 10uM Cisplatin for 24 h. Cell viability was then assessed using CellTiter-Glo Luminescent Cell Viability Assay and luminescence was measured using a Glomax Discover plates reader (Promega).

### Statistical analysis

Statistical differences were evaluated using Tukey’s multicomparison analysis after one-way ANOVA analysis to compare multiple samples or unpaired t test to compare only two samples using GraphPad Prism v9.0 (Graphpad Software, Inc., San Diego, California, USA).

## Results

### SMAD4 expression is higher in HPV-positive HNC cell lines compared to HPV-negative HNC cell lines and tumours

**Fig. 1:**
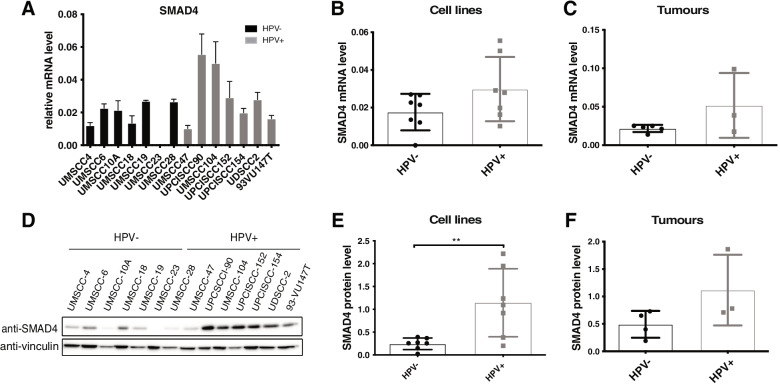
Analysis of SMAD4 expression in HPV-positive HNC cell lines and tumours. **A** Total RNAs from HNC cell lines were isolated for RT-qPCR. SMAD4 expression was normalized to RpP0 and shown as means ± SD of at least three independent experiments. **B** Box plot showing means ± SD and individual values of results shown in (**A**). **C** Total RNAs from frozen tissue samples of 5 HPV-negative and 3 HPV-positive HNC patients were isolated for RT-qPCR. SMAD4 expression was normalized to RpP0 and shown as Box plot of the means ± SD and individual values. **D** HNC cell lines were lysed and analysed by immunoblotting with the indicated antibodies. **E** Box plot showing means ± SD and individual values of Optical density analysis of the expression of SMAD4 normalised to loading control (vinculin) from at least three independent western blot experiments in HNC cell lines. **, *P* < 0.01 (unpaired t test). **F** Box plot showing means ± SD and individual values of Optical density analysis from immunoblotting of SMAD4 expression from frozen tissue samples of 4 HPV-negative and 3 HPV-positive HNC patients

Previous analysis of TGCA data [14], which we also confirmed (Fig. SA), showed that SMAD4 mRNA levels are higher in HPV-positive HNC compared to HPV-negative. We used a panel of 14 HNC cell lines 7 of which derived from HPV-positive and 7 from HPV-negative HNC and confirmed the greater expression of SMAD4 mRNA in HPV-positive HNC cell lines compared to the HPV-negative (Fig. 1A, B). Moreover, using tissue samples derived from HNC patients (5 HPV-negative and 3 HPV-positive) we substantiated that HPV-positive HNC showed greater expression of SMAD4 mRNA level compared to HPV-negative HNC (Fig. 1C). Interestingly, also SMAD4 protein is considerably more expressed in HPV-positive compared to HPV-negative HNC cell lines (Fig. 1D and E) and tumour samples (Fig. 1F and SB), even with a greater difference compared to SMAD4 mRNA level. All these data suggest that HPV might regulate SMAD4 expression.

### HPV16 regulates SMAD4 protein level

**Fig. 2:**
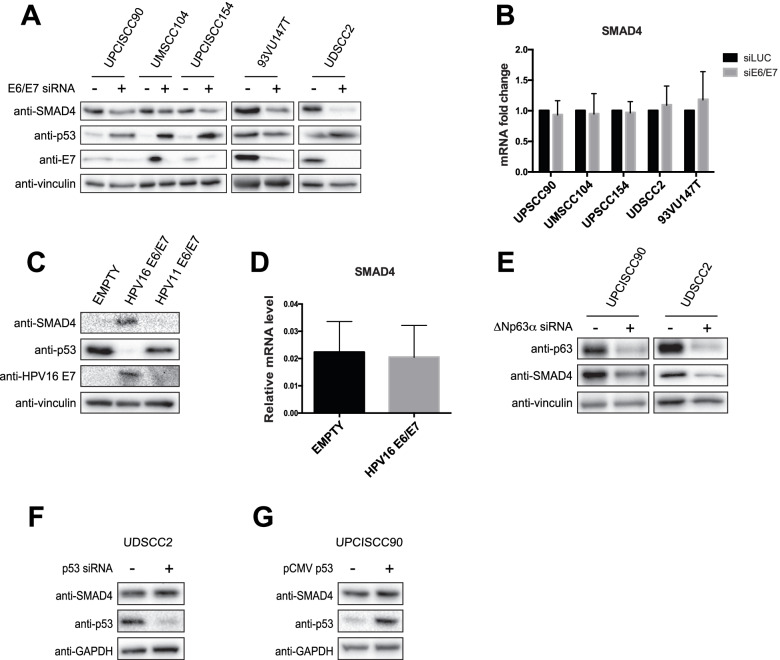
HPV16 regulation of SMAD4 expression. **A**, **B** HPV-positive HNC cell lines were transfected for 72 h with specific siRNA against HPV16 E6/E7 or Luciferase as control. After transfection cells were lysed and analysed by immunoblotting with the indicated antibodies (**A**) or total RNAs were isolated for RT-qPCR and reported as means ±SD of fold changes of at least three independent experiments (**B**). **C**, **D** HKs were transduced with empty or HPV16 E6/E7 or HPV11 E6/E7 recombinant retroviral vectors. After selection with G418 cells were harvested. **C** Lysates were collected and analysed by immunoblotting with the indicated antibodies. **D** Total RNAs were isolated for RT-qPCR. SMAD4 expression was normalized to RpP0. Results from at least three independent experiments are expressed as means ±SD. **E** HPV-positive cell lines were transfected with specific siRNA against ΔNp63α or Luciferase as control. 72 h after transfection cells were lysed and analysed by immunoblotting with the indicated antibodies. **F**, **G** HPV-positive HNC cell lines were transfected with either specific siRNA against p53 (**F**) or pCMV expressing p53 (**G**). Cells were the lysed and analysed by immunoblotting with the indicated antibodies

Thus, we hypothesised a direct regulation of SMAD4 by HPV16 E6/E7 oncoproteins. By modulation of E6/E7 expression upon knock-down in HNC cell lines or HPV16 E6/E7 retroviral transduction in primary human keratinocytes (HKs), we demonstrated a direct effect of high risk HPV16, but not low risk HPV11 on the expression of SMAD4 protein level (Fig. 2A and C respectively). The expression of HPV11 E6/E7 was verified by RT-qPCR (Fig. SC). Interestingly, the expression of SMAD4 mRNA was not affected by the modulation of E6/E7 expression (Fig. 2B and D), suggesting that high risk HPV modulates SMAD4 at post-transcriptional level. Even though SMAD4 mutation is not frequent in HNC, we verified whether the cell lines used were SMAD4 wt, using published data [26] and performing Sanger sequencing of SMAD4 exons for cell lines not yet tested (UMSCC10A, UMSCC23 and UMSCC28). As shown in Fig. 1A and D, only one HPV-positive cell line has low levels of SMAD4 mRNA and protein, the UMSCC47. This cell line does not expresses full-length p63, a member of the p53 family, due to the multiple integration of HPV16 at the *TP63* locus, leading to the expression of a truncated 25-kDa protein at the carboxyl terminus of p63 [27]. ΔNp63α, the most expressed isoform in HNC, is known to regulate SMAD4 expression in HPV-negative cell lines [28]. As shown in Fig. 2E, also in HPV-positive HNC cell lines ΔNp63α knock-down strongly reduces SMAD4 expression, suggesting that UMSCC47 has lower levels of SMAD4 compared to the other HPV-positive HNC cell lines due to the lack of ΔNp63α, whose expression is also regulated by HPV16 [19]. Since p53 silencing has been shown to increase SMAD4 expression in breast cancer cell lines [29], we hypothesise a possible role of p53, which is downregulated in the presence of E6, in the regulation of SMAD4. As Fig. 2F and G show, modulation of p53 by knock-down or overexpression did not change the expression of SMAD4, excluding a possible role of p53 in the regulation of SMAD4 expression.

### HPV stabilizes SMAD4 protein, interfering with its degradation

**Fig. 3:**
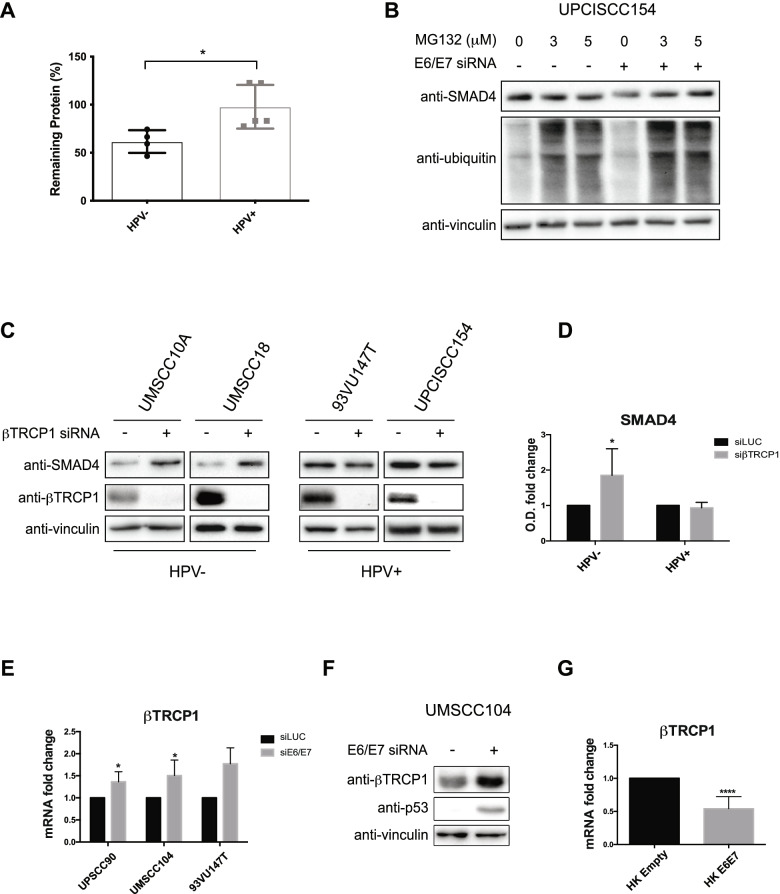
HPV16 regulation of SMAD4 degradation. **A** HPV-negative (UMSCC4, UMSCC6, UMSCC10A and UMSCC18) and HPV-positive (UMSCC47, UMSCC104, UPCISCC154, UDSCC2 and 93VU147T) HNC cell lines were treated with 10 μM Cycloheximide (CHX) for 16 h, lysed and analysed by immunoblotting. Results are represented as Box plot showing percentage of remaining protein ± SD after CHX treatment, calculated from Optical density analysis of the expression of SMAD4 normalized to loading control. *, *P* < 0.05 (unpaired t test). **B** UPCISCC154 were transfected with specific siRNA against HPV16 E6/E7 or Luciferase as control for 72 h and treated with two concentrations (3 or 5 μM) of MG132. Cells were then lysed and analysed by immunoblotting with the indicated antibodies. **C** HPV-positive (UPCISCC154 and 93VU147T) and HPV-negative (UMSCC10A and UMSCC18) HNC cell lines were transfected with specific siRNA against βTRCP1 or Luciferase. 72 h after transfection cells were lysed and analysed by immunoblotting with the indicated antibodies. **D** Graph shows the Optical density (O.D.) analysis of the expression of SMAD4 normalised to loading control (vinculin) and expressed as means ±SD of fold changes. *, *P* < 0.05 (Two-way Anova Multicomparison test) (**E**) HNC HPV-positive cell lines were transfected with specific siRNA against HPV16 E6/E7 or Luciferase as control. 72 h after transfection total RNAs were isolated for RT-qPCR and reported as means ±SD of fold changes. *, *P* < 0.05 (unpaired t test) (**F**) UMSCC104 cell line was transfected with specific siRNA against HPV16 E6/E7 or Luciferase as control. 72 h after transfection cells were lysed and analysed by immunoblotting with the indicated antibodies. **G** HKs were transduced with empty or HPV16 E6/E7 recombinant retroviral vectors. After selection with G418 cells total RNAs were isolated for RT-qPCR. βTRCP1 expression was normalized to RpP0. Results from at least three independent experiments are expressed as means ±SD of fold changes. ****, *P* < 0.0001 (unpaired t test)

We then assessed the stability of SMAD4 protein by cycloheximide (CHX) treatment. As shown in Fig. 3A, SMAD4 half-life is longer in HPV-positive HNC cell lines compared to the HPV-negative. Thus, HPV seems to stabilize SMAD4 protein and this result was confirmed in UPCISCC154 cells by the use of the proteasome inhibitor MG132 which completely reverted the reduction of SMAD4 protein induced by the lack of E6/E7 (Fig. 3B). The degradation of SMAD4 seems to be mediated by different ubiquitin E3 ligases; in particular βTRCP1 was shown to induce the degradation of SMAD4 upon a phosphorylation cascade [30]. We thus assessed whether this ligase was involved in the degradation of SMAD4 in our system. By knocking down βTRCP1, we observed increased levels of SMAD4 protein only in HPV-negative and not in HPV-positive HNC cell lines (Fig. 3C), suggesting that βTRCP1 promotes SMAD4 ubiquitination preferentially in HPV-negative HNC cell lines. Notably, HPV seems to be directly involved in the regulation of βTRCP1 expression since E6/E7 knock-down or HPV16 E6/E7 retroviral transduction modulated the expression of this ligase (Fig. 3E/F and 3G respectively). All these data suggest that lower expression of βTRCP1, due to the presence of HPV, might be involved in the increased stability of SMAD4.

### βTRCP1 is less expressed in HPV-positive HNC and is less efficient in ubiquitinating SMAD4

**Fig. 4:**
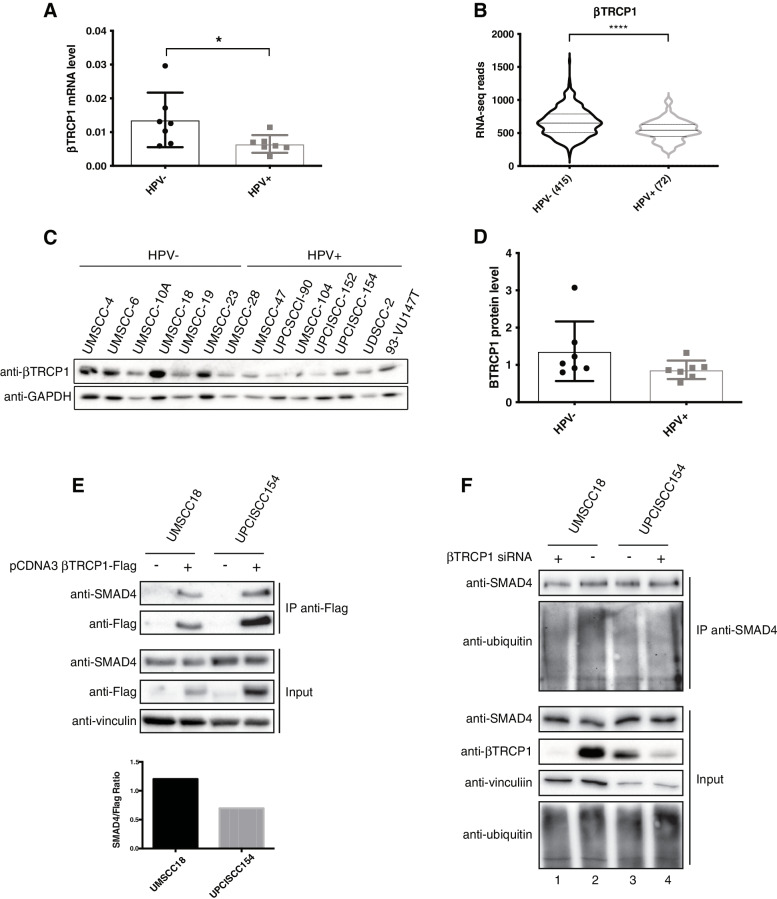
Analysis of βTRCP1-dependent SMAD4 degradation in HPV-negative and HPV-positive HNC cell lines. **A** Total RNAs from HNC cell lines were isolated for RT-qPCR. βTRCP1 expression was normalized to RpP0 and shown as Box plot of means ± SD and individual values of at least three independent experiments. *, *P* < 0.05 (unpaired t test). **B** Normalized RNA-seq data were extracted from TCGA database and divided in HPV+ and HPV- HNC cohorts. Numbers in brackets refer to the number of samples included in each analysis. ****, *P* < 0.0001 (unpaired t test). **C** HNC cell lines were lysed and analysed by immunoblotting with the indicated antibodies. **D** Box plot showing means ± SD and individual values of Optical density analysis of the expression of βTRCP1 normalised to loading control (GAPDH) from three independent western blot experiments in HNC cell lines. **E** pcDNA3-βTRCP1-flag plasmid was transfected into HPV-positive (UPCISCC154) and HPV-negative (UMSCC18) HNC cell lines. One day after transfection cell were treated with 5 μM MG132 overnight. Two days after transfection cells were harvested and whole cells extracts were subjected to anti-Flag immunoprecipitation (IP). Immunoblotting with the indicated antibodies was then performed and whole cell extracts were used as input controls. Graph shows Optical density analysis of SMAD4 co-immunoprecipitated normalised to βTRCP1-flag immunoprecipitated. **F** HPV-positive (UPCISCC154) and HPV-negative (UMSCC18) HNC cell lines were transfected with specific siRNA against βTRCP1 or Luciferase. Two days after transfection cell were treated with 5 μM MG132 overnight. 72 h after transfection cells were harvested and whole cells extracts were subjected to anti-SMAD4 immunoprecipitation (IP). Immunoblotting with the indicated antibodies was then performed and whole cell extracts were used as input controls

We then analysed βTRCP1 expression in HNC cell lines and human tumours. As Fig. 4A and B show, in both HNC cell lines and tumours βTRCP1 mRNA is greatly expressed in HPV-negative compared to HPV-positive samples, results also confirmed at protein level in HNC cell lines (Fig. 4C, D). All these data suggest that the lower expression of βTRCP1 in HPV-positive HNC cell lines might explain the higher expression of SMAD4. We then showed by co-immunoprecipitation, that βTRCP1 had lower affinity in binding SMAD4 in HPV-positive compared to HPV-negative cell lines (Fig. 4E), and that lack of βTRCP1 reduced SMAD4 ubiquitination only in HPV-negative and not in HPV-positive cell lines (Fig. 4F compare lane 1 and 2). These data confirm that βTRCP1 preferentially binds and ubiquitinates SMAD4 in HPV-negative compared to HPV-positive HNC cell lines.

### The expression of the DDR protein CHK1 and Rad51 highly correlates with SMAD4 expression in HNC cell lines and is modulated by HPV16

**Fig. 5:**
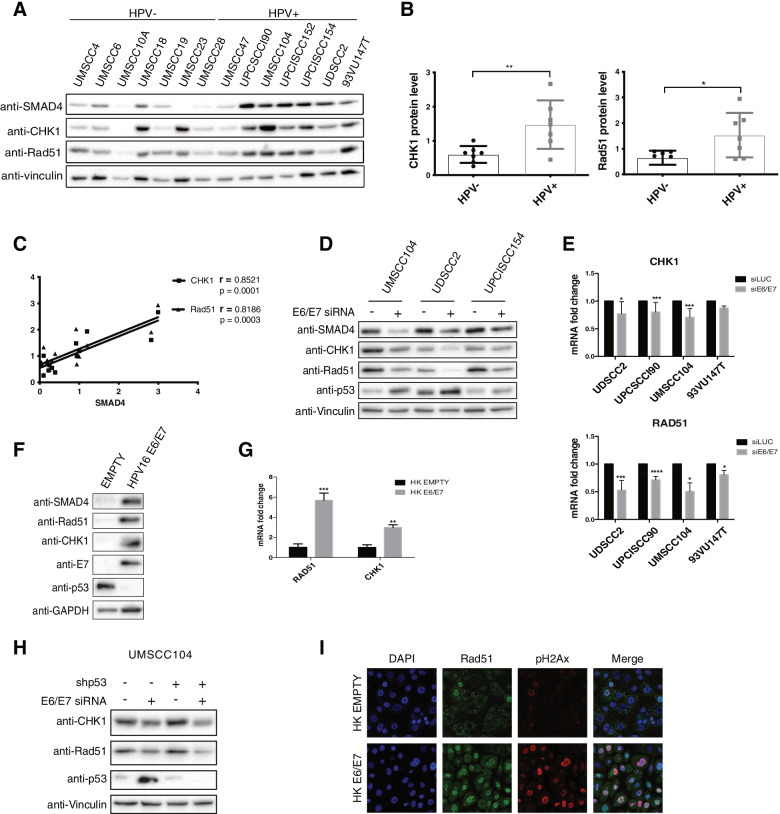
HPV regulation of CHK1 and Rad51 expression. **A** HNC cell lines were lysed and analysed by immunoblotting with the indicated antibodies. **B** Box plot showing means ± SD and individual values of Optical density analysis of the expression of CHK1 and Rad51 normalised to loading control (vinculin) from at least three independent western blot experiments in HNC cell lines. *, *P* < 0.05; **, *P* < 0.01 (unpaired t test). **C** Results from Optical density analysis of SMAD4 in HNC cell lines were correlated to CHK1 and Rad51 and Pearson correlation coefficient (r) was calculated. **D**, **E** HPV-positive HNC cell lines were transfected for 72 h with specific siRNA against HPV16 E6/E7 or Luciferase as control. After transfection cells were lysed and analysed by immunoblotting with the indicated antibodies (**D**) or total RNAs were isolated for RT-qPCR and reported as means ±SD of fold changes of at least three independent experiments (**E**). **F**, **G** HKs were transduced with empty or HPV16 E6/E7 recombinant retroviral vectors. After G418 selection, cells were harvested. **F** Lysates were collected and analysed by immunoblotting with the indicated antibodies. **G** Total RNAs were isolated for RT-qPCR. SMAD4 expression was normalized to RpP0. Results from at least three independent experiments are expressed as means ±SD of fold changes. **, *P* < 0.01; ***, *P* < 0.001 (unpaired t test). **H** UMSCC104 cells were transduced with shp53 or control. After antibiotic selection, cells were transfected for 72 h with specific siRNA against HPV16 E6/E7 or Luciferase as control. After transfection cells were lysed and analysed by immunoblotting with the indicated antibodies. **I** HKs were transduced with empty or HPV16 E6/E7 recombinant retroviral vectors. After G418 selection, cells were seeded on coverslips and after 24 h fixed and stained with the indicated antibodies for Immunofluorescence. Coverslips were mounted and visualized using a Leica SP8 AOBS confocal microscopy

HPV is known to modulate the DDR response to promote its life-cycle [31]. SMAD4 mRNA levels correlate with the expression of some DDR genes [6, 32], pointing to a possible signature role for SMAD4 similar to BRCA1 in breast cancer. Figure 5A and B clearly showed that two of the main proteins involved in the homologous recombination (HR) response, Checkpoint kinase 1 (CHK1) and Rad51 recombinase, were greatly expressed in HPV-positive compared to HPV-negative HNC cell lines, and that SMAD4 expression highly correlates with the expression of these two genes in HNC cell lines (Fig. 5C). These data were confirmed also upon analysing the TCGA dataset (Fig. SD) and assessing the expression of these proteins in HNC tissue samples. Indeed, CHK1 and Rad51 expression was greater in HPV-positive compared to HPV-negative HNC tissue samples (Figs. SB, SE and SF). These results confirm the importance of SMAD4 in maintaining genomic stability in HNC.

Moreover**,** upon modulating E6/E7 expression either through knock-down in HNC cell lines (Fig. 5D and E) or HPV16E6/E7 retroviral transduction in HKs (Fig. 5F and G), the expression of SMAD4, Rad51 and CHK1, as well as of other genes involved in the DDR response, was modified (Fig. SG, SH). Interestingly, differently from SMAD4, also Rad51 and CHK1 mRNA was affected by HPV modulation (Fig. 5E and G), suggesting a different mechanism of regulation of these two genes with respect to SMAD4. Also in this case, as for SMAD4, p53 was not involved in the regulation of the expression of these DDR genes (Fig. 5H). Interestingly, HPV16-dependent upregulation of Rad51 was linked to and increased recruitment of this protein to the chromatin. Indeed, upon HPV16 transduction in HKs, there was an increased number and colocalization of both Rad51 and pH2Ax foci in the nucleus (Fig. 5I). All these data suggest that HPV16 control the DNA damage response upregulating the expression of genes involved in the repair pathway.

### SMAD4 regulates DDR gene expression and is responsible for the HPV-dependent regulation of the DD response

**Fig. 6:**
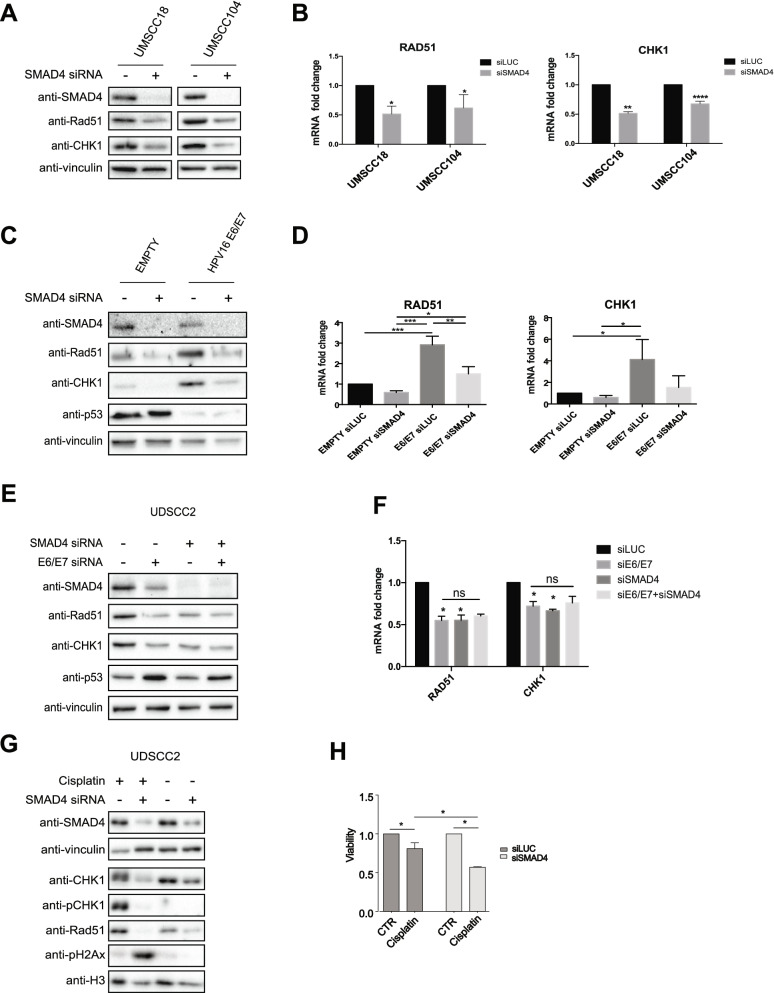
Analysis of HPV modulation of DD response upon SMAD4 silencing. **A**, **B** HPV-positive (UMSCC104) and HPV-negative (UMSCC18) HNC cell lines were transfected with specific siRNA against SMAD4 or Luciferase as control. 72 h after transfection cells were lysed and analysed by immunoblotting with the indicated antibodies (**A**) or total RNAs were isolated for RT-qPCR and reported as means ±SD of fold changes of at least three independent experiments. *, *P* < 0.05; **, *P* < 0.01; ****, *P* < 0.0001 (unpaired t test) (**B**). **C**, **D** HKs were transduced with empty or HPV16 E6/E7 recombinant retroviral vectors. After selection with G418 cells were transfected with specific siRNA against SMAD4 or Luciferase as control. 72 h after transfection cells were lysed and analysed by immunoblotting with the indicated antibodies (**C**) or total RNAs were isolated for RT-qPCR and reported as means ±SD of fold changes of three independent experiments. *, *P* < 0.05; **, *P* < 0.01; ***, *P* < 0.001 (One-way Anova Multicomparison test) (**D**). **E**, **F** UDSCC2 cell line was transfected with specific siRNA against SMAD4 and HPV16 E6/E7 alone or in combination, or Luciferase as control. 72 h after transfection cells were lysed and analysed by immunoblotting with the indicated antibodies (**E**) or total RNAs were isolated for RT-qPCR and reported as means ±SD of fold changes over siLUC. *, *P* < 0.05, ns, not significant (One-way Anova Multicomparison test) (**F**). **G** UDSCC2 cell line was transfected with specific siRNA against SMAD4 or Luciferase as control. 48 h after transfection cells were treated for 24 h with 10 μM Cisplatin and subsequently lysed and analysed by immunoblotting with the indicated antibodies. **H** UDSCC2 cell line was transfected with specific siRNA against SMAD4 or Luciferase as control. 24 h after transfection cells were seeded into 96well plates (7000 cells/well) and 24 h later were treated with 10 μM Cisplatin for 24 h. Cell viability was then assessed using CellTiter-Glo® Luminescent Cell Viability Assay and expressed as viability relative to untreated cells (means ±SD of fold changes) of two independent experiments. *, *P* < 0.05; (One-way Anova Multicomparison test)

SMAD4 was shown to regulate DDR genes [6] and lack of SMAD4 reduces DDR gene expression and induces genomic instability. As Fig. 6A and B show, SMAD4 knock-down led to the reduction of Rad51 and CHK1 at both protein and mRNA level, suggesting a transcriptional regulation mediated by SMAD4. Thus, since HPV activates DDR and upregulates SMAD4 expression, we wondered whether SMAD4 was acting downstream of HPV16 in the regulation of DNA damage response. To prove this hypothesis, we transduced HKs with HPV16 E6/E7 retroviral vector and subsequently, we knocked-down SMAD4. As Fig. 6C and D show, the upregulation of Rad51 and CHK1 mediated by HPV was abolished upon SMAD4 knock-down. These results were also validated by knocking-down both SMAD4 and E6/E7 alone or in combination in the HPV-positive HNC cell line UDSCC2. Figure 6E and F show that by single knock-down of both SMAD4 and HPV in UDSCC2 cell line, the reduction of Rad51 and CHK1 was similar, and did not increase in the double knock-down, suggesting that HPV and SMAD4-dependent regulation of these DDR genes are on the same pathway. Finally, in UDSCC2 cells cisplatin treatment upon SMAD4 silencing (Fig. 6G) increased CHK1 phosphorylation and partially H2Ax phosphorylation, suggesting an activation of the HR machinery. Moreover, the lack of SMAD4 induced a greater accumulation of pH2Ax, implying a defect in repairing the damage, which might be due to the lack of expression of the DDR genes promoted by the absence of SMAD4. Finally, lack of SMAD4 significantly decreased the viability of UDSCC2 cells upon cisplatin treatment (Fig. 6H), suggesting that SMAD4 protein levels might affect the activation of the DD response and as a consequence be responsible of the treatment efficacy.

## Discussion

TGFβ is well-known for its anti-proliferative activity and malignant progression is typically associated with the loss of responsiveness to TGFβ-induced cell growth inhibition. The cell-cycle machinery of proliferating cells is necessary for HPV replication. TGFβ profoundly suppresses epithelial cell proliferation. Studies in cervical cancer show that HPV-encoded proteins bind to TGFβ receptors and signal transducers, resulting in their degradation and promoting the proliferation of infected cells [33]. Interestingly, we have previously shown [18] that in both HPV-positive and negative HNC cell lines the TGFβ pathway is regularly activated and promotes transcription of mesenchymal genes. In particular, one of the main player of the TGFβ pathway SMAD4, whose expression is usually decreased in many tumours such as HNC, has been previously shown to be upregulated in HPV-positive HNC tumours compared to HPV-negative [14]. Moreover, genetic alterations at the SMAD4 locus such as deletions or mutations, are much rare in HPV-positive compared to HPV-negative HNC [14, 26]. In contrast, despite HPV-positive HNC and cervical carcinomas are both driven by HPV infection, cervical cancer samples showed a high frequency (41%) of LOH at SMAD4 gene locus [34] and similar data were also reported in cervical cancer cell lines [35]. In this study, although in a limited number of HNC samples, we confirmed the upregulation of SMAD4 mRNA in HPV-positive HNC tumours compared to HNC-negative and we show that also at protein level the expression of SMAD4 is higher in HPV-positive HNC. All these data were completely reproduced in a panel of HNC cell lines, showing how the use of these cell lines perfectly recapitulates the characteristic of the tumours. Moreover, we found that HPV16-encoded oncoproteins regulate SMAD4 expression at protein but not at mRNA level, without involving the action of p53. Nevertheless, another component of the p53 family, p63, is involved in the regulation of SMAD4 expression in HPV-positive HNC cell lines, explaining the lower SMAD4 level in one of the HPV-positive HNC cell lines used, the UMSCC47, which does not express the full-length ΔNp63α isoform, due to multiple integration of HPV16 at the TP63 locus [27]. Interestingly, HPV16 affects the stability of SMAD4 protein by reducing its degradation. Many viruses and pathogens, and in particular the papillomaviruses, have been implicated in the regulation of posttranscriptional pathways such ubiquitination and SUMOylation, affecting the expression of the enzymes involved in these pathways [36, 37]. In particular, *Listeria monocytogenes* impairs the SUMO pathway, thus decreasing the stability of SMAD4 [38], which is stabilized by SUMOylation [39]. Here we show how HPV16 is responsible for the downregulation of the expression of one E3 ubiquitin ligase, the βTRCP1, which has been previously shown to induce SMAD4 ubiquitination and degradation. βTRCP1 leads to SMAD4 degradation upon sequential phosphorylation mediated by GSK3 and mitogen-activated protein kinase (MAPK) [30]. Here we show how βTRCP1 preferentially degrades SMAD4 in HPV-negative and not in HPV-positive HNC cell lines, where SMAD4 is less ubiquitinated and has lower affinity to binding βTRCP1. One of the explanations of the different affinity of βTRCP1 to bind SMAD4, which differentiates HPV-negative and HPV-positive HNC cell lines, might be due to a different activation of the phosphorylation events that affects the binding of βTRCP1 to SMAD4 and its subsequent degradation. SMAD4 degradative defects in HPV-positive cell lines might be also explained by the fact that HPV downregulates βTRCP1 expression, leading to a lower expression of this ligase in HPV-positive HNC cell lines and tumours compared to HPV-negative. The ubiquitin pathway is not the only pathway influenced by viruses; the DNA repair pathway is a target of many viruses since DDR proteins play a role in the cellular response to viral infection, as well as in the lifecycle of many viruses. HPV both inhibits and activates different elements of the DDR pathway. This double behaviour has led to discrepancy in the interpretation of data studying the effect of HPV infection on the DDR machinery, which seems to be linked to a higher radiosensitivity of HPV-dependent tumours. In particular, the homologous recombination has been shown to be both inhibited [40, 41] and activated by HPV16 oncoproteins [42]. CHK1 is one of the key downstream molecules of DDR signalling. In response to DNA damage, CHK1 is phosphorylated primarily by ATR kinase [43], leading to cell cycle arrest in S and at G2/M phases and promoting DNA repair before cell division. CHK1 phosphorylates many substrate proteins including the recombinase RAD51, the central molecule in HR pathway, that binds single-strand DNA at the sites of damage and forms filaments that are observed microscopically as nuclear foci. Here we show how HPV16, is able to upregulate the expression of both CHK1 and Rad51, together with the expression of other genes involved in the DDR signalling. This HPV-dependent upregulation leads to the increased expression of these proteins in both HNC tumours and cell lines and we also demonstrated that these two enzymes are still active in the presence of the virus. Indeed, upon cisplatin treatment in UDSCC2 cells, CHK1 is regularly phosphorylated and, in HKs transduced with HPV16 E6/E7, Rad51 foci increase compared to empty control cells and colocalize with pH2Ax, suggesting that Rad51 activity is maintained.

SMAD4 expression is deeply linked to the expression of genes involved in the DNA damage response [6] and regulates the response to genotoxic [6] and DNA repair inhibitors [32]. In particular, here we highlight how both HNC cell lines and tumours recapitulate what shown upon interrogating the TCGA dataset [32]. SMAD4 expression truly correlates with the expression of some genes involved in the ATR activation of the HR response, such as Rad51 and CHK1, and this is due to a transcriptional regulation mediated by SMAD4. Even more interestingly, we found that HPV-mediated upregulation of the DDR genes is dependent on the presence of SMAD4. Thus, as previously suggested in cervical cancer model [44], HPV oncogenes promote initiation of the HR pathway but somehow impede the complete repair of the damage. This leads to an increased number of pH2Ax foci and might explain the greater sensitivity to radiotherapy of HPV-positive HNC tumours compared to the HPV-negative ones. Since HPV needs HR proteins to promote its replication, we hypothesize that the virus increases SMAD4 expression to upregulate HR and facilitate its replication. Finally, we show that in HPV-positive cell lines SMAD4-loss sensitizes cells to DNA-damaging agents, such as cisplatin, suggesting that SMAD4-deficient HPV tumours may be preferentially susceptible to similar treatments.

## Conclusions

Our data provide a rationale for the establishment of a new signature associated to SMAD4 expression in HNC, and in particular in HPV-positive HNC. Detection of SMAD4 levels can be used as a predictive marker to select patients that would benefit from new targeted therapies, based on DNA repair checkpoint inhibitors alone or in combination with DNA-damaging agent, to increase therapy efficacy, de-escalate cisplatin treatment and reduce the incidence of treatment resistant tumours.

## Supplementary Information

**Additional file 1.**

## Data Availability

All relevant data are within the paper and its Supporting Information files.

## Abbreviations

HPV: Human Papillomavirus
HNC: Head and Neck cancer
DDR: DNA damage repair
TGF-β: Transforming growth factor- β
HKs: Human keratinocytes
LOH: Loss of heterozygosity
CHX: Cycloheximide
CHK1: Checkpoint kinase 1
TCGA: The Cancer Genome Atlas Program

## References

[1] Siegel PM, Massagué J. Cytostatic and apoptotic actions of TGF-β in homeostasis and cancer. Vol. 3, Nature Reviews Cancer. 2003. p. 807–20. 10.1038/nrc1208 14557817

[2] Malkoski SP, Wang XJ. Two sides of the story? Smad4 loss in pancreatic cancer versus head-and-neck cancer. Vol. 586, FEBS Letters. 2012. p. 1984–92. 10.1016/j.febslet.2012.01.054 PMC3285395 22321641

[3] Haeger SM, Thompson JJ, Kalra S, Cleaver TG, Merrick D, Wang XJ, et al. Smad4 loss promotes lung cancer formation but increases sensitivity to DNA topoisomerase inhibitors. Oncogene. 2016;35(5):577–86. 10.1038/onc.2015.112 PMC4615192 25893305

[4] Liu J, Cho SN, Akkanti B, Jin N, Mao J, Long W, et al. ErbB2 pathway activation upon smad4 loss promotes lung tumor growth and metastasis. Cell Rep. 2015;10(9):1599–613. 10.1016/j.celrep.2015.02.014 PMC7405934 25753424

[5] Tascilar M, Rosty C, Wilentz RE, Kern SE, Hruban RH, Goggins M, et al. The SMAD4 protein and prognosis of pancreatic ductal adenocarcinoma. Clin Cancer Res. 2001;7(12):4115–21. 11751510

[6] Bornstein S, White R, Malkoski S, Oka M, Han G, Cleaver T, et al. Smad4 loss in mice causes spontaneous head and neck cancer with increased genomic instability and inflammation. J Clin Invest. 2009;119(11):3408–19. 10.1172/JCI38854 PMC2769185 19841536

[7] Izeradjene K, Combs C, Best M, Gopinathan A, Wagner A, Grady WM, et al. KrasG12D and Smad4/Dpc4 Haploinsufficiency Cooperate to Induce Mucinous Cystic Neoplasms and Invasive Adenocarcinoma of the Pancreas. Cancer Cell. 2007;11(3):229–43. 10.1016/j.ccr.2007.01.017 17349581

[8] Bardeesy N, Cheng KH, Berger JH, Chu GC, Pahler J, Olson P, et al. Smad4 is dispensable for normal pancreas development yet critical in progression and tumor biology of pancreas cancer. Genes Dev. 2006;20(22):3130–46. 10.1101/gad.1478706 PMC1635148 17114584

[9] Ding Z, Wu CJ, Chu GC, Xiao Y, Ho D, Zhang J, et al. SMAD4-dependent barrier constrains prostate cancer growth and metastatic progression. Nature. 2011;470(7333):269–76. 10.1038/nature09677 PMC3753179 21289624

[10] Yang L, Mao C, Teng Y, Li W, Zhang J, Cheng X, et al. Targeted disruption of Smad4 in mouse epidermis results in failure of hair follicle cycling and formation of skin tumors. Cancer Res. 2005;65(19):8671–8. 10.1158/0008-5472.CAN-05-0800 16204035

[11] Hunter KD, Parkinson EK, Harrison PR. Profiling early head and neck cancer. Vol. 5, Nature Reviews Cancer. 2005. p. 127–35. 10.1038/nrc1549 15685196

[12] Leemans CR, Snijders PJF, Brakenhoff RH. The molecular landscape of head and neck cancer. Vol. 18, Nature Reviews Cancer. 2018. p. 269–82. 10.1038/nrc.2018.11 29497144

[13] Takebayashi S, Ogawa T, Jung KY, Muallem A, Mineta H, Fisher SG, et al. Identification of new minimally lost regions on 18q in head and neck squamous cell carcinoma. Cancer Res. 2000;60(13):3397–403. 10910046

[14] Cheng H, Fertig EJ, Ozawa H, Hatakeyama H, Howard JD, Perez J, et al. Decreased SMAD4 expression is associated with induction of epithelial-to-mesenchymal transition and cetuximab resistance in head and neck squamous cell carcinoma. Cancer Biol Ther. 2015;16(8):1252–8. 10.1080/15384047.2015.1056418 PMC4623002 26046389

[15] Gillespie KA, Mehta KP, Laimins LA, Moody CA. Human Papillomaviruses Recruit Cellular DNA Repair and Homologous Recombination Factors to Viral Replication Centers. J Virol. 2012;86(17):9520–6. 10.1128/JVI.00247-12 PMC3416172 22740399

[16] Reinson T, Toots M, Kadaja M, Pipitch R, Allik M, Ustav E, et al. Engagement of the ATR-Dependent DNA Damage Response at the Human Papillomavirus 18 Replication Centers during the Initial Amplification. J Virol. 2013;87(2):951–64. 10.1128/JVI.01943-12 PMC3554080 23135710

[17] Brenner JC, Graham MP, Kumar B, Saunders LM, Kupfer R, Lyons RH, et al. Genotyping of 73 UM-SCC head and neck squamous cell carcinoma cell lines. Head Neck. 2010;32(4):417–26. 10.1002/hed.21198 PMC3292176 19760794

[18] Citro S, Bellini A, Miccolo C, Ghiani L, Carey TE, Chiocca S. Synergistic antitumour activity of HDAC inhibitor SAHA and EGFR inhibitor gefitinib in head and neck cancer: a key role for ΔNp63α. Br J Cancer. 2019;120(6):658–67. 10.1038/s41416-019-0394-9 PMC6461861 30765872

[19] Citro S, Bellini A, Medda A, Sabatini ME, Tagliabue M, Chu F, et al. Human Papilloma Virus Increases ΔNp63α Expression in Head and Neck Squamous Cell Carcinoma. Front Cell Infect Microbiol. 2020;10:143. 10.3389/fcimb.2020.00143 PMC7156594 32322564

[20] Pozzebon ME, Varadaraj A, Mattoscio D, Jaffray EG, Miccolo C, Galimberti V, et al. BC-box protein domain-related mechanism for VHL protein degradation. Proc Natl Acad Sci U S A. 2013;110(45):18168–73. 10.1073/pnas.1311382110 PMC3831490 24145437

[21] Mattoscio D, Casadio C, Miccolo C, Maffini F, Raimondi A, Tacchetti C, et al. Autophagy regulates UBC9 levels during viral-mediated tumorigenesis. PLoS Pathog. 2017;13(3): e1006262. 10.1371/journal.ppat.1006262 PMC5349695 28253371

[22] Busino L, Donzelli M, Chiesa M, Guardavaccaro D, Ganoth D, Dorrello NV, et al. Degradation of Cdc25A by β-TrCP during S phase and in response to DNA damage. Nature. 2003;426(6962):87–91. 10.1038/nature02082 14603323

[23] Baker SJ, Fearon ER, Nigro JM, Hamilton SR, Preisinger AC, Jessup JM, et al. Chromosome 17 deletions and p53 gene mutations in colorectal carcinomas. Science. 1989;244(4901):217–21. 10.1126/science.2649981 2649981

[24] Cerami E, Gao J, Dogrusoz U, Gross BE, Sumer SO, Aksoy BA, et al. The cBio Cancer Genomics Portal: An open platform for exploring multidimensional cancer genomics data. Cancer Discov. 2012;2(5):401–4. 10.1158/2159-8290.CD-12-0095 PMC3956037 22588877

[25] Gao J, Aksoy BA, Dogrusoz U, Dresdner G, Gross B, Sumer SO, et al. Integrative analysis of complex cancer genomics and clinical profiles using the cBioPortal. Sci Signal. 2013;6(269). 10.1126/scisignal.2004088 PMC4160307 23550210

[26] Kalu NN, Mazumdar T, Peng S, Shen L, Sambandam V, Rao X, et al. Genomic characterization of human papillomavirus-positive and -negative human squamous cell cancer cell lines. Oncotarget. 2017;8(49):86369–83. 10.18632/oncotarget.21174 PMC5689691 29156801

[27] Akagi K, Li J, Broutian TR, Padilla-Nash H, Xiao W, Jiang B, et al. Genome-wide analysis of HPV integration in human cancers reveals recurrent, focal genomic instability. Genome Res. 2014;24(2):185–99. 10.1101/gr.164806.113 PMC3912410 24201445

[28] Calleja LR, Jacques C, Lamoureux F, Baud’Huin M, Gabriel MT, Quillard T, et al. ΔNp63α silences a miRNA program to aberrantly initiate a wound-healing program that promotes TGFβ-induced metastasis. Cancer Res. 2016;76(11):3236–51. 10.1158/0008-5472.CAN-15-2317 PMC4891241 26988989

[29] Wu B, Li W, Qian C, Zhou Z, Xu W, Wu J. Down-Regulated P 53 by siRNA increases Smad4’s activity in promoting cell apoptosis in MCF-7 cells. Eur Rev Med Pharmacol Sci. 2012;16(9):1243–8. 23047509

[30] Demagny H, Araki T, de Robertis EM. The tumor suppressor Smad4/DPC4 is regulated by phosphorylations that integrate FGF, Wnt, and TGF-β signaling. Cell Rep. 2014;9(2):688–700. 10.1016/j.celrep.2014.09.020 25373906

[31] Graham S V. The human papillomavirus replication cycle, and its links to cancer progression: A comprehensive review. Vol. 131, Clinical Science. 2017. p. 2201–21. 10.1042/CS20160786 28798073

[32] Hernandez AL, Young CD, Bian L, Weigel K, Nolan K, Frederick B, et al. PARP inhibition enhances radiotherapy of SMAD4-deficient human head and neck squamous cell carcinomas in experimental models. Clin Cancer Res. 2020;26(12):3058–70. 10.1158/1078-0432.CCR-19-0514 PMC7299799 32139402

[33] Pérez-Plasencia C, Vázquez-Ortiz G, López-Romero R, Piña-Sanchez P, Moreno J, Salcedo M. Genome wide expression analysis in HPV16 Cervical Cancer: Identification of altered metabolic pathways. Infect Agent Cancer. 2007;2(1). 10.1186/1750-9378-2-16 PMC2034543 17822553

[34] Kloth JN, Kenter GG, Spijker HS, Uljee S, Corver WE, Jordanova ES, et al. Expression of Smad2 and Smad4 in cervical cancer: Absent nuclear Smad4 expression correlates with poor survival. Mod Pathol. 2008;21(7):866–75. 10.1038/modpathol.2008.62 18425078

[35] Baldus SE, Schwarz E, Lohrey C, Zapatka M, Landsberg S, Hahn SA, et al. Smad4 deficiency in cervical carcinoma cells. Oncogene. 2005;24(5):810–9. 10.1038/sj.onc.1208235 15531914

[36] Citro S, Chiocca S. Listeria monocytogenes: A bacterial pathogen to hit on the SUMO pathway. Cell Res. 2010;20(7):738–40. 10.1038/cr.2010.76 20531377

[37] Mattoscio D, Medda A, Chiocca S. Recent highlights: Onco viral exploitation of the sumo system. Curr Issues Mol Biol. 2020;35:1–16. 10.21775/cimb.035.001 31422930

[38] Ribet D, Hamon M, Gouin E, Nahori MA, Impens F, Neyret-Kahn H, et al. Listeria monocytogenes impairs SUMOylation for efficient infection. Nature. 2010;464(7292):1192–5. 10.1038/nature08963 PMC3627292 20414307

[39] Lin X, Liang M, Liang YY, Brunicardi FC, Feng XH. SUMO-1/Ubc9 promotes nuclear accumulation and metabolic stability of tumor suppressor Smad4. J Biol Chem. 2003;278(33):31043–8. 10.1074/jbc.C300112200 12813045

[40] Dok R, Kalev P, Van Limbergen EJ, Asbagh LA, Vázquez I, Hauben E, et al. P16INK4a impairs homologous recombination-mediated DNA repair in human papillomavirus-positive head and neck tumors. Cancer Res. 2014;74(6):1739–51. 10.1158/0008-5472.CAN-13-2479 24473065

[41] Weaver AN, Cooper TS, Rodriguez M, Trummell HQ, Bonner JA, Rosenthal EL, et al. DNA double strand break repair defect and sensitivity to poly ADP-ribose polymerase (PARP) inhibition in human papillomavirus 16-positive head and neck squamous cell carcinoma. Oncotarget. 2015;6(29):26995–7007. 10.18632/oncotarget.4863 PMC4694969 26336991

[42] Leeman JE, Li Y, Bell A, Hussain SS, Majumdar R, Rong-Mullins X, et al. Human papillomavirus 16 promotes microhomology-mediated end-joining. Proc Natl Acad Sci U S A. 2019;116(43):21573–9. 10.1073/pnas.1906120116 PMC6815166 31591214

[43] Goto H, Kasahara K, Inagaki M. Novel insights into chk1 regulation by phosphorylation. Cell Struct Funct. 2014;40(1):43–50. 10.1247/csf.14017 25748360

[44] Wallace NA, Khanal S, Robinson KL, Wendel SO, Messer JJ, Galloway DA. High-Risk Alphapapillomavirus Oncogenes Impair the Homologous Recombination Pathway. J Virol. 2017;91(20). 10.1128/JVI.01084-17 PMC5625488 28768872

