# Supplementary material for: HPV-mediated regulation of SMAD4 modulates the DNA damage response in head and neck cancer
Source: J Exp Clin Cancer Res. 2022 Feb 10;41:59. doi: 10.1186/s13046-022-02258-9 (PMC8830113; doi:10.1186/s13046-022-02258-9)
Supplement: Supplementary file 1 — Additional file 1. [file 13046_2022_2258_MOESM1_ESM.pdf]

Figure S

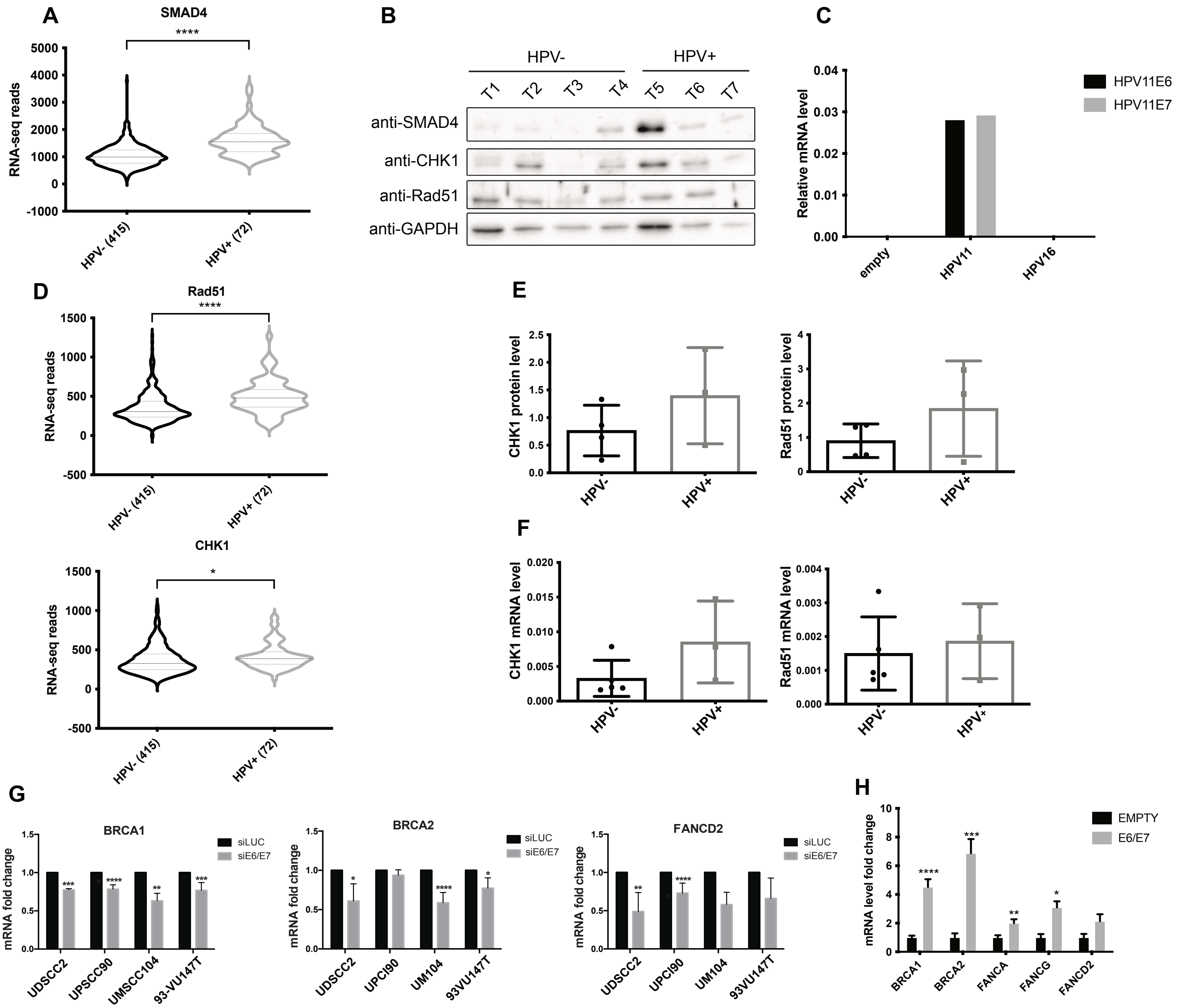

Figure S. (A) Normalized RNA-seq data for SMAD4 were extracted from the TCGA PanCancer Atlas and divided in HPV+ and HPV- HNC cohorts. Numbers in brackets refer to the number of samples included in each analysis. ****, $P < 0.0001$ (unpaired t test). (B) Protein fraction was extracted from frozen tissue samples of 4 HPV-negative and 3 HPV-positive HNC patients and analysed by immunoblotting with the indicated antibodies. (C) HKs were transduced with empty or HPV16 E6/E7 or HPV11 E6/E7 recombinant retroviral vectors. After selection with G418 cells were harvested total RNAs were isolated for RT-qPCR. HPV11 E6/E7 expression was normalized to RpP0. (D) Normalized RNA-seq data for SMAD4 was extracted from the TCGA PanCancer Atlas and divided in HPV+ and HPV- HNC cohorts. Numbers in brackets refer to the number of samples included in each analysis. *, $P < 0.05$; ****, $P < 0.0001$ (unpaired t test). (E) Box plot showing means ± SD and individual values Optical density analysis of the expression of CHK1 and Rad51 from frozen tissue samples of 4 HPV-negative and 3 HPV-positive HNC patients. (F) Total RNAs from frozen tissue samples of 5 HPV-negative and 3 HPV-positive HNC patients were isolated for RT-qPCR. CHK1 and Rad51 expression was normalized to RpP0 and shown as Box plot of the means ± SD and individual values. (G) HNC HPV-positive cell lines were transfected with specific siRNA against HPV16 E6/E7 or Luciferase as control. 72h after transfection cells total RNAs were isolated for RT-qPCR and reported as means ±SD of fold changes of at least three independent experiments. (H) HKs were transduced with empty or HPV16 E6/E7 recombinant retroviral vectors. After selection with G418 cells were harvested total RNAs were isolated for RT-qPCR. DDR gene expression was normalized to RpP0. Results from at least three independent experiments are expressed as means ±SD of fold changes. **, $P < 0.01$; ***, $P < 0.001$; ****, $P < 0.0001$ (unpaired t test).
